# Effects of Typical Underground Coal Mine Environmental Factors on CO Oxidation Performance of Sn-Containing Catalyst

**DOI:** 10.3390/molecules31050838

**Published:** 2026-03-02

**Authors:** Tianyu Xin, Bing Liang, Jiaxu Jin, Gang Bai, Junguang Wang, Qiang Liu, Yashengnan Sun, Xihua Zhou

**Affiliations:** 1 School of Safety Science and Engineering, Liaoning Technical University, Huludao 125100, China; 2 School of Mechanics and Engineering, Liaoning Technical University, Fuxin 123000, China; 3 School of Architecture and Construction, Liaoning Technical University, Fuxin 123000, China

**Keywords:** Sn-containing catalyst, CO catalytic oxidation, coal mine safety, water poisoning, temperature effect

## Abstract

One of the primary causes of casualties as a result of underground coal mine disasters is the generation of high concentrations of carbon monoxide (CO). In this study, a copper (Cu)–manganese (Mn)–tin (Sn) composite oxide catalyst was prepared using the co-precipitation method, and the effects of CO concentration (1–7%), reaction temperature (25–300 °C), and water poisoning degree (0–100%) on CO catalytic oxidation performance were systematically investigated using a dynamic activity testing system. The results demonstrated that within the CO concentration range of 1–7%, the catalyst was able to reduce the CO concentration to below 0.55% in a maximum of 248 s and maintain this level in a relatively stable state. Meanwhile, both the catalytic activity and maximum instantaneous reaction rate exhibited a linear increase with the rise in the CO concentration. Elevated temperature significantly shortened the equilibrium time and reduced the equilibrium concentration, achieving 99.99% elimination efficiency at 300 °C; however, catalyst activity decreased with increasing temperature due to adsorption step limitations. Water poisoning severely affected catalyst performance, with activity, elimination efficiency, and long-term stability exhibiting exponential decay as the water poisoning degree increased, with the most significant performance decline occurring in the 0–60% range. Based on the dynamic gas concentration analysis, the CO oxidation process with this catalyst exhibited characteristics consistent with the Mars–van Krevelen mechanism. These findings provide an experimental basis for evaluating the applicability of Sn-containing catalysts in extreme underground coal mine environments.

## 1. Introduction

In the event of underground coal mine disasters, including gas explosions, coal dust explosions, and mine fires, substantial amounts of carbon monoxide (CO) gas are generated, posing severe threats to miners’ lives [1,2,3]. Statistics indicate that CO concentrations following gas explosions can reach 7–8%, with approximately 70–80% of casualties in underground accidents attributed to CO poisoning [4,5]. The affinity of CO for hemoglobin is approximately 200–250 times greater than that of oxygen, leading to severe hypoxia and potentially death [6,7]. Therefore, developing efficient and reliable CO emergency elimination technologies is of great significance for ensuring coal miners’ safety.

Currently, common CO elimination methods include cryogenic separation, chemical absorption, pressure swing adsorption, and catalytic oxidation [8,9,10]. Among these, catalytic oxidation can efficiently convert CO to non-toxic CO_2_ under mild conditions, offering advantages such as moderate reaction conditions, no secondary pollution, and continuous operation, making it the most promising CO elimination technology [11,12,13]. Although precious metal catalysts (Pt, Pd, Au, etc.) exhibit excellent low-temperature activity, their high costs limit their use in large-scale applications [14,15,16]. Transition metal oxide catalysts have attracted widespread attention due to their low cost and abundant resources. Among them, Cu-Mn composite oxides (hopcalite catalysts) have been industrially applied in respiratory protection and other fields, owing to their excellent room-temperature CO oxidation activity [17,18,19]. However, these catalysts are prone to significant activity loss in environments with relative humidity above 40% due to the competitive adsorption of H_2_O molecules, severely limiting their practical application in high-humidity underground coal mines [20,21].

To enhance the water resistance of Cu-Mn catalysts, researchers have conducted various modification studies. Dey et al. [22] found that doping with Fe or Ni significantly improved low-temperature activity; Yang et al. [23] reported that mesoporous Cu-Mn oxides show excellent room-temperature catalytic performance; and Ma et al. [24] demonstrated that appropriate Sn doping can maintain high activity while significantly enhancing material stability in humid environments. Additionally, doping with Ce, Co, Ag, and other elements has been proven to improve catalyst resistance to water poisoning [25,26,27].

Despite significant progress detailed in the aforementioned studies, existing work has primarily employed low CO concentrations (<1%) and idealized conditions for testing, with the systematic evaluation of catalyst performance under complex operating conditions remaining relatively scarce. Underground coal mine disaster environments possess unique characteristics: the CO concentration can rapidly increase to several percent, local temperatures can rise to hundreds of degrees Celsius, and relative humidity typically remains at high levels (>80%) [28,29]. A systematic investigation of the synergistic effects of a high CO concentration, wide temperature range, and different humidity conditions in terms of the performance of a modified Cu-Mn catalyst remains scarce [30].

To fill this research gap, for the present study, we selected a copper (Cu)–manganese (Mn)–tin (Sn) composite oxide catalyst with excellent potential water resistance as the research object and systematically investigated the effects of different CO concentrations (1–7%), reaction temperatures (25–300 °C), and water poisoning levels (0–100%) on its CO catalytic oxidation performance. This study focused on analyzing the reaction process characteristics, activity variation patterns, instantaneous reaction rates, removal efficiency, and long-term stability of the catalyst, while also exploring its catalytic reaction mechanism. This research provides an experimental basis for clarifying the environment–structure–performance relationship of the copper–manganese–tin composite oxide catalyst and offers data support for its practical application in underground coal mine environments.

## 2. Results and Discussion

### 2.1. Catalyst Optimization and Selection

**Figure 1:**
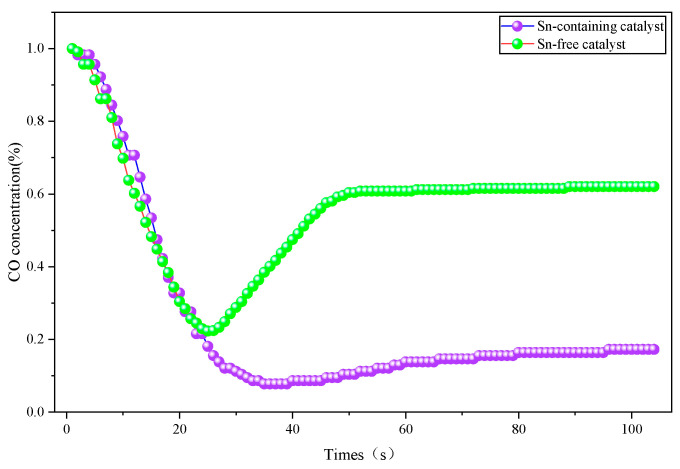
Curves of CO concentration versus time during the ablation process of different samples in a dry environment.

**Figure 2:**
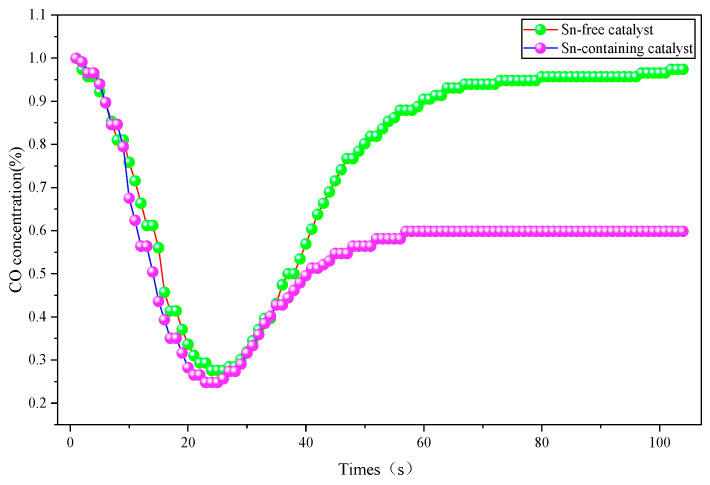
Curves of CO concentration versus time during the ablation process of different samples in a humid environment.

The Sn-free catalyst and Sn-containing catalyst were synthesized following the method detailed in Section 2.1. Their catalytic activity and water resistance were evaluated separately under dry and humid environmental conditions, with the activity test results presented in Figure 1 and Figure 2.

As indicated in Figure 1, the CO concentration variation curves show two distinct inflection points, corresponding to the concentration minimum and the equilibrium point, respectively. Under dry conditions, the Sn-containing catalyst maintained a CO concentration at a much lower level and achieved a state of relative equilibrium, reducing the initial CO concentration from 1% to approximately 0.12%. In comparison, the Sn-free catalyst only reduced the CO concentration from 1% to around 0.61% and maintained that relatively stable level. These results demonstrate that the catalytic activity of the Sn-containing catalyst is significantly superior to that of the Sn-free catalyst in dry environments.

Figure 2 illustrates the time-dependent variation in the CO concentration during the ablation process of various samples under humid conditions. It can be observed that the catalytic activity of both catalysts decreased in the humid environment. Specifically, the Sn-free catalyst was nearly completely deactivated. In contrast, although the Sn-containing catalyst also experienced a certain loss of activity, it still retained favorable efficiency for the catalytic oxidation of CO. A compelling piece of evidence is that it reduced the CO concentration from 1% to approximately 0.58% and maintained this relatively stable level. These findings confirm that Sn doping is beneficial for enhancing the ablation performance of the ablative agent in humid environments.

In subsequent experiments, based on the Sn-containing catalyst, test conditions featuring high temperatures, high humidity, and a high CO concentration will be established to simulate the potential environmental conditions in underground coal mines. The practical applicability of the Sn-containing catalyst in such underground coal mine settings will be further analyzed.

### 2.2. Effect of CO Concentration on Catalytic Performance

#### 2.2.1. Reaction Process Analysis

**Figure 3:**
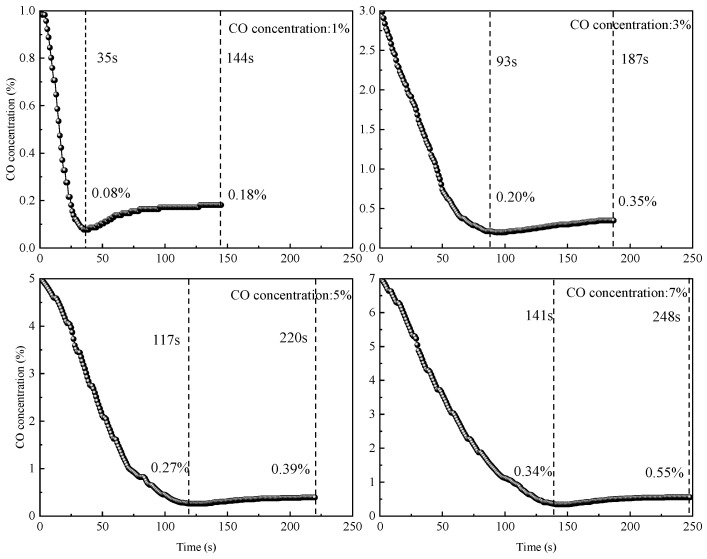
CO concentration variation curves during catalyst reaction under different gas concentration conditions.

To investigate the effect of different CO concentrations on catalytic reaction processes in underground coal mine conditions, dynamic elimination tests were conducted with mixed gases containing 1%, 3%, 5%, and 7% CO. CO concentration variations over time during the reaction were recorded using a gas analyzer, and the results are shown in Figure 3.

As shown in Figure 3, the CO concentration variation trends during the catalyst reaction were generally similar under different CO concentration conditions. In the early reaction stage, the CO concentration decreased to a minimum point, followed by a slight increase, and finally approached catalytic oxidation equilibrium. Compared with the 1% CO concentration, the times to reach minimum concentration points under 3%, 5%, and 7% conditions were delayed by 58 s, 82 s, and 106 s, respectively, with the minimum concentration points increased by 1.5%, 2.38%, and 3.25%. Compared with low-CO-concentration conditions, a high CO concentration caused the minimum and equilibrium concentration points to shift toward longer reaction times and higher CO concentrations, with the degree of shift increasing with CO concentration. This occurs because the number of active sites on the catalyst surface is limited; when the CO concentration is too high, the number of CO molecules exceeds the active site’s capacity, causing some CO molecules to pass through the catalyst without being captured, resulting in elevated minimum concentration points. When the CO concentration was below 7%, the catalyst could reduce the CO concentration to below 0.55% within 248 s, providing effective escape time for underground personnel.

When the CO concentrations were 1%, 3%, 5%, and 7%, the catalytic oxidation equilibrium times were 144 s, 187 s, 220 s, and 248 s, respectively, with equilibrium time extending as the CO concentration increased. This is because as the CO concentration increases, the number of CO molecules flowing over the catalyst surface increases correspondingly, meaning that a longer amount of time is needed to reach reaction equilibrium. Simultaneously, the equilibrium concentration also increased with CO concentration, with catalytic oxidation equilibrium concentrations of 0.18%, 0.35%, 0.39%, and 0.55%, respectively. This is because an elevated CO concentration causes CO molecules to be in an oversaturated state relative to active sites, with some unadsorbed CO molecules directly entering the gas analyzer, causing an elevated equilibrium concentration.

**Figure 4:**
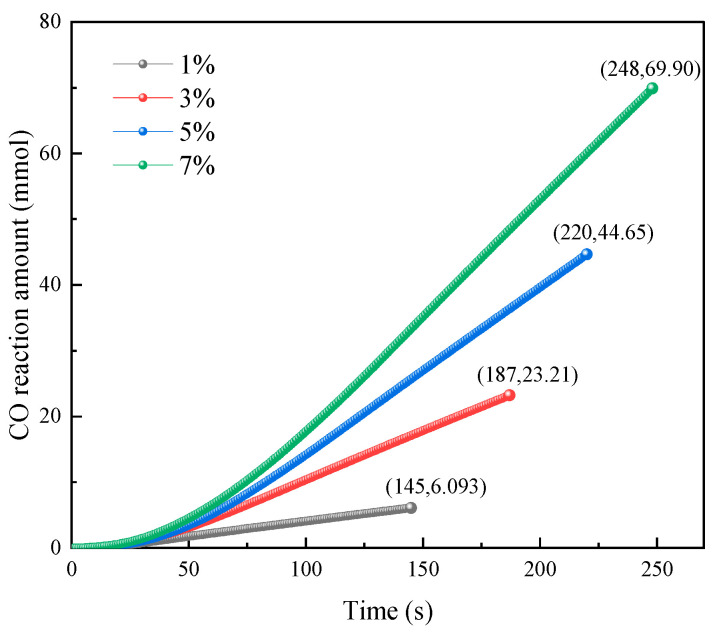
Variation in the CO reaction amount with time.

**Figure 5:**
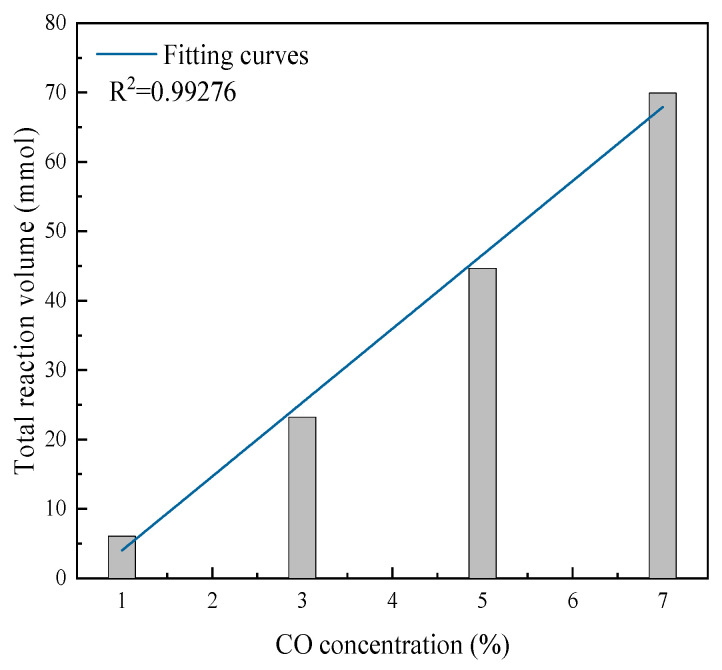
Total reaction amount at different gas concentrations.

To quantify the total CO conversion and dynamic variation patterns during the reaction, the CO reaction amount versus time curves and total reaction amount versus CO concentration curves were plotted, as shown in Figure 4 and Figure 5.

As shown in Figure 4, the temporal trends of the CO reaction amount were generally similar under different CO concentration conditions, with relatively slow growth in the early reaction period and nearly linear growth in the later period. Throughout the reaction process, the CO reaction amounts consistently followed the order of 1% < 3% < 5% < 7%. This is because at low CO concentrations, the number of CO molecules in the mixed gas is small, resulting in insufficient CO molecule supply relative to the number of catalyst surface active sites. As CO concentration increased, more active sites were occupied by CO molecules, and the reaction amounts gradually increased.

As shown in Figure 5, the total CO reaction amount increased linearly with the CO concentration. At CO concentrations of 1%, 3%, 5%, and 7%, the corresponding total reaction amounts were 6.09 mmol, 23.22 mmol, 44.66 mmol, and 69.90 mmol, respectively. This is because at lower CO concentrations, although not all CO molecules could be eliminated, the catalyst surface active sites remained in excess relative to CO molecules. As the CO concentration increased, more catalyst surface active sites became occupied, thus increasing the total CO reaction amount.

#### 2.2.2. Effect of CO Concentration on Catalyst Activity

**Figure 6:**
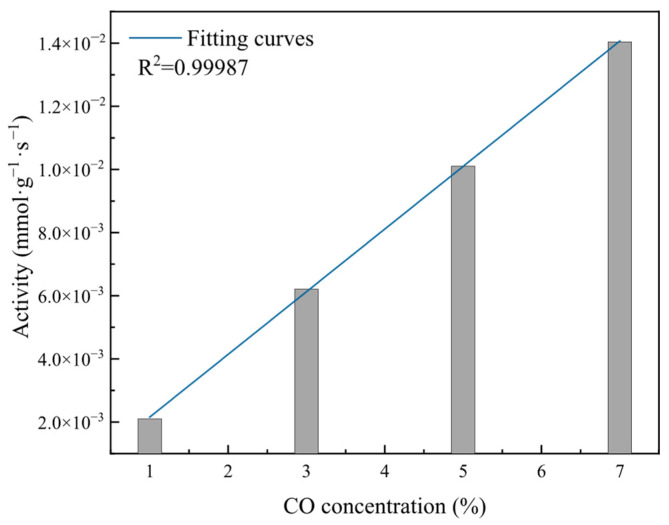
Effect of different CO concentrations on catalyst activity during reaction.

**Figure 7:**
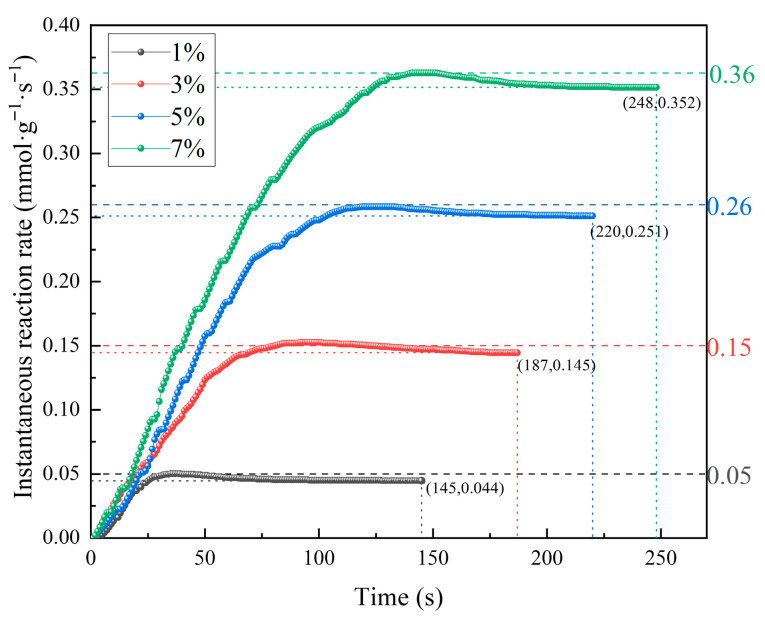
Variation in the instantaneous reaction rate with time during catalyst elimination at different gas concentrations.

To clarify the effect of changes in the CO concentration on catalyst activity, the total catalyst activity and instantaneous reaction rates at different CO concentrations were calculated according to Equations (7) and (9), and the results are shown in Figure 6 and Figure 7.

As shown in Figure 6, at CO concentrations of 1%, 3%, 5%, and 7%, the catalyst activities were 2.1 × 10^−3^ mmol·g^−1^·s^−1^, 6.21 × 10^−3^ mmol·g^−1^·s^−1^, 1.01 × 10^−2^ mmol·g^−1^·s^−1^, and 1.40 × 10^−2^ mmol·g^−1^·s^−1^, respectively. The catalyst activity increased linearly with the increasing CO concentration. This is because the catalyst activity represents the total rate of the catalytic reaction; an increased CO concentration increases the number of reactant molecules, thereby increasing the forward reaction rate and causing the catalyst activity during the elimination process to increase linearly with the CO concentration.

As shown in Figure 7, the instantaneous reaction rate versus time curves exhibited step-like patterns, indicating that the CO concentration significantly affected the instantaneous reaction rates during the reaction, with higher CO concentrations in the mixed gas corresponding to higher instantaneous reaction rates. This is because at higher CO concentrations, more CO molecules flow over the catalyst surface, and an increased reactant concentration accelerates the catalytic oxidation reaction rate. At mixed gas concentrations of 1%, 3%, 5%, and 7%, the maximum instantaneous rates were 0.05 mmol·g^−1^·s^−1^, 0.15 mmol·g^−1^·s^−1^, 0.26 mmol·g^−1^·s^−1^, and 0.36 mmol·g^−1^·s^−1^, respectively. This is because the reaction rate is proportional to the reactant concentration [31]; therefore, the maximum instantaneous reaction rate increases with the increase in the CO concentration.

### 2.3. Effect of Reaction Temperature on Catalytic Performance

#### 2.3.1. Reaction Process Analysis

**Figure 8:**
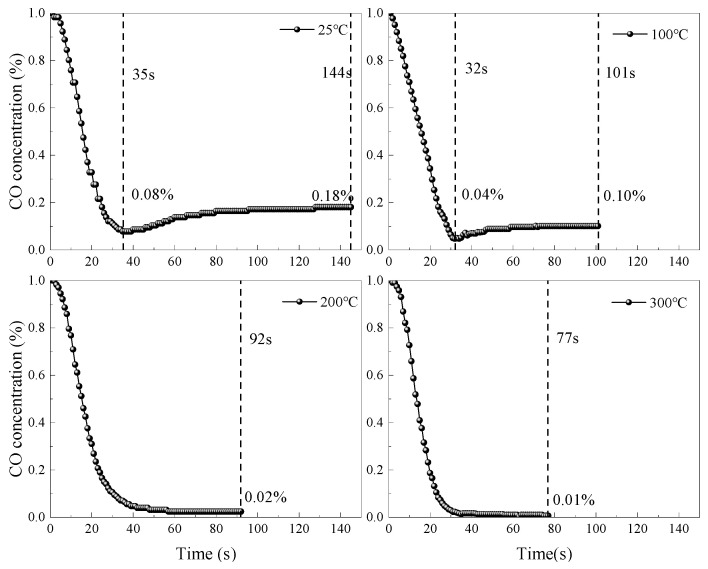
Variation in the CO concentration during catalyst reaction under different temperature conditions.

To explore the effect of underground coal mine temperature on catalyst elimination processes, CO concentration versus time data during elimination were collected using a gas analyzer under conditions of 1% CO concentration and 19.8% O_2_ concentration, and the results are shown in Figure 8.

As shown in Figure 8, when mixed gas flowed through the catalyst, the CO concentration decreased rapidly. At temperatures of 25 °C and 100 °C, the CO concentration curves showed slow increases after reaching minimum points, eventually reaching equilibrium. However, at temperatures of 200 °C and 300 °C, the CO concentration curves reached equilibrium concentration directly after reaching minimum points without any increase. The inflection point in CO concentration curves occurs because both CO and O_2_ molecules chemisorb on the catalyst surface [18,32]. At reaction onset, CO reacts with active oxygen species to form CO_2_, causing the CO concentration to decrease rapidly to the minimum point. As the reaction proceeds, the generated CO_2_ does not completely desorb, occupying some CO active sites, leading to reduced CO adsorption sites and a slight increase in the CO concentration before reaching equilibrium. At 200 °C and 300 °C, no inflection points appeared because high temperature promotes CO_2_ desorption from active sites, facilitating CO adsorption and elimination, thus reaching elimination equilibrium directly without inflection points.

Comparing the CO concentration variation curves under room-temperature conditions, high temperatures shortened the elimination process, with higher temperatures corresponding to shorter catalyst elimination times. At temperatures ≥ 300 °C, the catalyst could reduce the 1% CO concentration to 0.01% within 77 s. Further comparison of the changes in the CO concentration under room- and high-temperature conditions revealed that the CO concentration inflection points disappeared at temperatures > 200 °C, indicating that elevated temperature is beneficial for CO elimination using the catalyst.

At temperatures of 25 °C, 100 °C, 200 °C, and 300 °C, the catalyst equilibrium times were 144 s, 101 s, 92 s, and 77 s, with equilibrium concentrations of 0.18%, 0.10%, 0.02%, and 0.01%, respectively. Compared with room-temperature conditions (25 °C), high-temperature (300 °C) conditions reduced the elimination equilibrium time by approximately 46% and decreased the equilibrium concentration by 94% (from 0.18% to 0.01%), indicating that the catalyst is more suitable for high-temperature catalytic oxidation and can eliminate CO more thoroughly in high-temperature environments. This phenomenon occurs because elevated temperatures intensify molecular motion, accelerating the catalytic oxidation reaction process and thus reaching equilibrium earlier. Simultaneously, an increase in temperature causes mixed gas thermal expansion, reducing the number of gas molecules per unit volume, allowing CO to be more completely adsorbed on the catalyst surface. Since adsorption is the first step of catalytic oxidation, complete CO adsorption further promotes catalytic oxidation, resulting in a lower equilibrium concentration.

**Figure 9:**
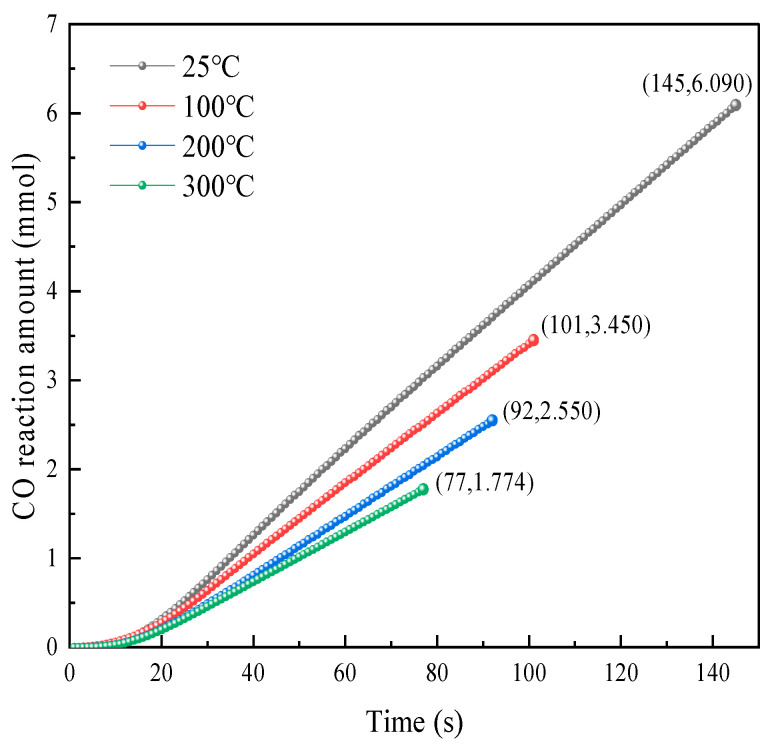
Variation in the CO reaction amount with time at different temperatures.

**Figure 10:**
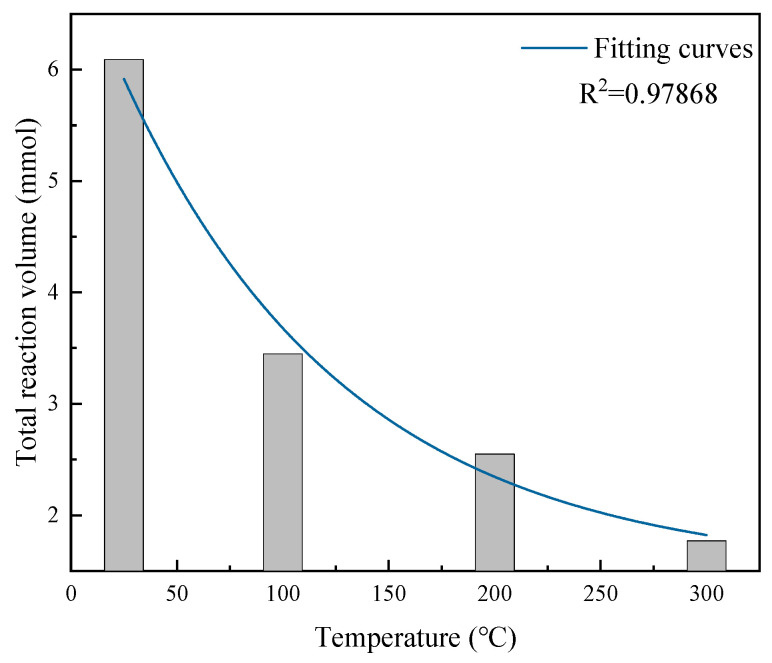
Total elimination reaction amount at each temperature.

To further analyze the CO reaction amounts during reactions under different temperature conditions, the CO reaction amount versus time curves and total CO reaction amount versus temperature curves were plotted, as shown in Figure 9 and Figure 10.

As shown in Figure 9, the variation trends of the CO reaction over time were generally similar at different temperatures, with relatively slow growth in the CO reaction amount in the early reaction period. This is mainly because in the initial reaction stage, after CO adsorbs on the catalyst surface’s active sites, it must undergo adsorption–activation–reaction processes, resulting in slower growth in the CO reaction amount at the initial stage. As the reaction proceeded, the catalytic oxidation reaction on the catalyst surface gradually reached a steady state, at which point the CO reaction amount increased linearly with time. Whether in the initial or later stages of the reaction, the CO reaction amount at the same time point consistently followed the order of 25 °C > 100 °C > 200 °C > 300 °C. According to the ideal gas law, as temperature increases, the molar amount of mixed gas flowing through the catalyst decreases, correspondingly reducing the CO reaction amount.

As shown in Figure 10, at temperatures of 25 °C, 100 °C, 200 °C, and 300 °C, the total CO reaction amounts were 6.09 mmol, 3.45 mmol, 2.55 mmol, and 1.77 mmol, respectively. The total CO reaction amount during the reaction exhibited exponential decay with increasing temperature. This is because as temperature increases, gas expands, reducing the number of CO and O_2_ molecules in the same volume of mixed gas. Additionally, higher temperatures result in shorter equilibrium times, and since the flow rate remains constant during experiments, shorter times mean that less mixed gas flows through the catalyst. The combination of these two factors leads to a reduction in the total reaction amount, causing the total reaction amount to decay exponentially with increasing temperature. From a reaction timeliness perspective, temperature increase enhances catalyst elimination performance. Simultaneously, higher temperatures result in lower catalytic oxidation equilibrium concentrations, indicating that temperature increase promotes CO catalytic oxidation.

#### 2.3.2. Effect of Reaction Temperature on Catalyst Activity

**Figure 11:**
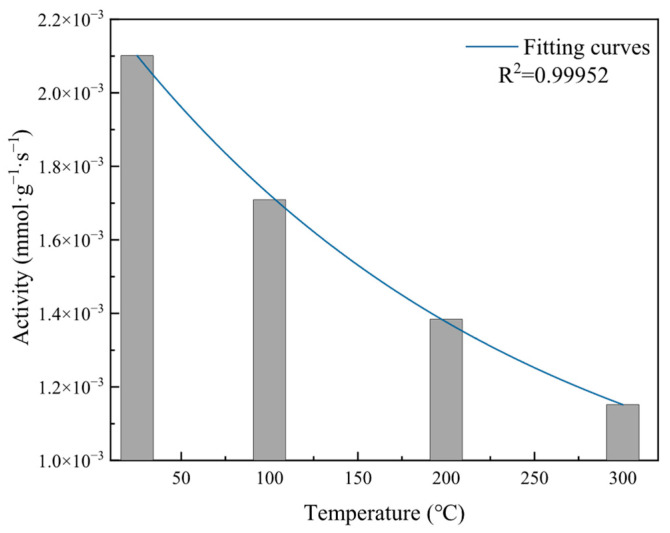
Effect of different temperatures on catalyst activity during reaction.

**Figure 12:**
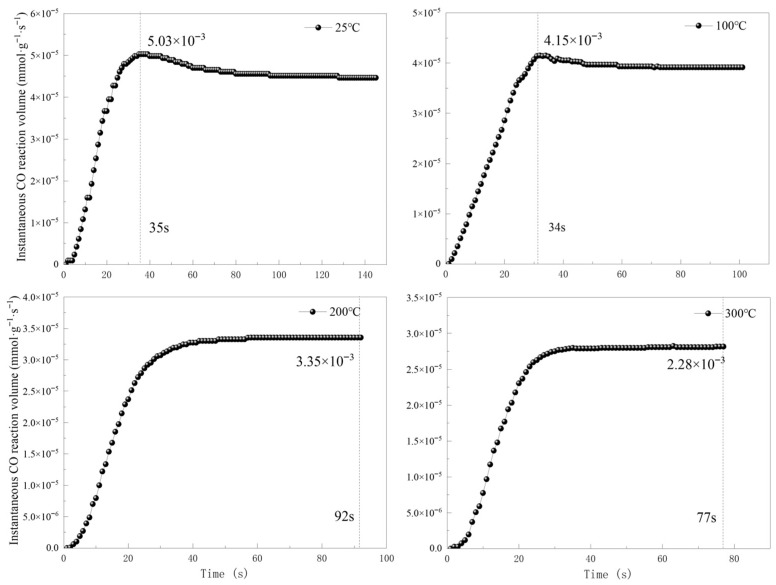
Variation in the instantaneous reaction rate with time under different temperature conditions.

To investigate the effect of temperature on catalyst activity, the total catalyst activity and instantaneous reaction rates at different temperatures were calculated and plotted, as shown in Figure 11 and Figure 12.

As shown in Figure 11, at temperatures of 25 °C, 100 °C, 200 °C, and 300 °C, the activities were 2.10 × 10^−3^ mmol·g^−1^·s^−1^, 1.71 × 10^−3^ mmol·g^−1^·s^−1^, 1.38 × 10^−3^ mmol·g^−1^·s^−1^, and 1.15 × 10^−3^ mmol·g^−1^·s^−1^, respectively. The sample’s total activity exhibited exponential decay with increasing temperature. From this, the CO elimination process over the catalyst can be divided into three steps: CO and O_2_ chemisorption; reaction of CO and O_2_ on the catalyst surface; and desorption of CO_2_ formed by the reaction. Under the present experimental conditions, the adsorption step was the rate-controlling step. Although temperature increase accelerated surface reaction rates, it inhibited CO and O_2_ adsorption processes, causing adsorption rates to decrease and thus the total reaction rate (i.e., activity) to decrease with increasing temperature.

As shown in Figure 12, at 25 °C and 100 °C, the instantaneous reaction rates increased rapidly in the early reaction period, reaching maximum instantaneous rates before showing slight decreases; after this, the curves leveled off, finally reaching catalytic oxidation equilibrium. At 200 °C and 300 °C, instantaneous reaction rates similarly increased rapidly in the early period but directly entered catalytic oxidation equilibrium after reaching peak values without rate decrease stages. This difference stems from the selective effects of temperature increase on reaction steps. Temperature increase favors CO and O_2_ reaction, increasing reaction rates, while simultaneously inhibiting catalyst adsorption and activation rates for CO and O_2_. This causes CO and O_2_ adsorption rates to quickly reach dynamic equilibrium with CO oxidation reaction rates; therefore, at 200 °C and 300 °C, instantaneous reaction rates directly approach equilibrium after reaching their peaks.

The maximum instantaneous reaction rates at 25 °C, 100 °C, 200 °C, and 300 °C were 5.03 × 10^−3^, 4.15 × 10^−3^, 3.35 × 10^−3^, and 2.28 × 10^−3^ mmol·g^−1^·s^−1^, respectively. The maximum instantaneous reaction rate decreased with increasing temperature. The reason for this is that although an increase in temperature can enhance the reaction rates of CO and O_2_, the inhibitory effect on CO and O_2_ adsorption processes is more significant. As the rate-controlling step, the decreased adsorption rate dominated the trend of decreasing the maximum instantaneous reaction rate with increasing temperature.

#### 2.3.3. Effect of Temperature on Catalyst Elimination Efficiency

**Figure 13:**
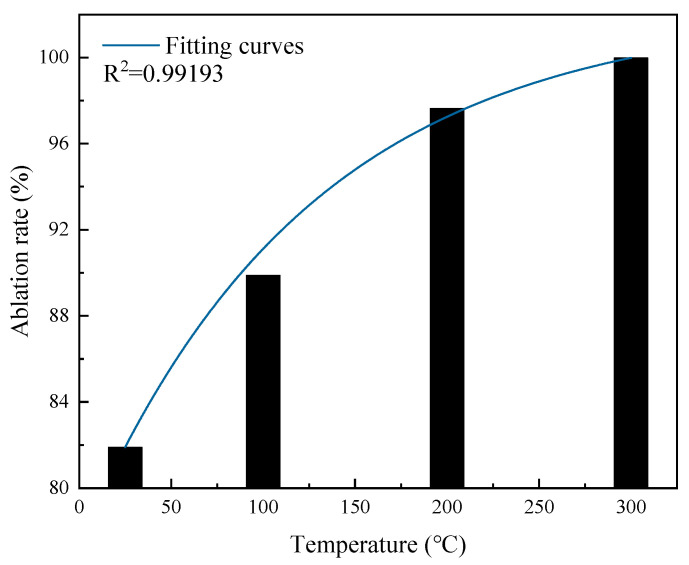
Variation in the elimination efficiency with temperature.

The catalyst elimination efficiency during the reaction was calculated using Equation (10), and the results are plotted in Figure 13.

As shown in Figure 13, at temperatures of 25 °C, 100 °C, 200 °C, and 300 °C, the corresponding elimination efficiencies were 81.90%, 89.90%, 97.64%, and 99.99%, respectively. The elimination efficiency exhibited an exponential growth trend with increasing temperature. When the ambient temperature was ≥300 °C, the elimination efficiency reached 99.99% and significantly improved compared to room temperature (25 °C), indicating that elevated temperature is beneficial for improving the catalyst’s elimination efficiency. Under the premise that the mixed gas flow rate remains constant, the number of gas molecules flowing through the catalyst at the same time decreases with increasing temperature. When the gas molecule numbers decrease, the catalyst surface active site adsorption efficiency for CO and O_2_ molecules is more easily enhanced, thereby promoting catalytic oxidation reactions and ultimately causing catalyst elimination efficiency to significantly increase with temperature.

### 2.4. Effect of Water Poisoning on Catalyst Performance

#### 2.4.1. Reaction Process Analysis

**Figure 14:**
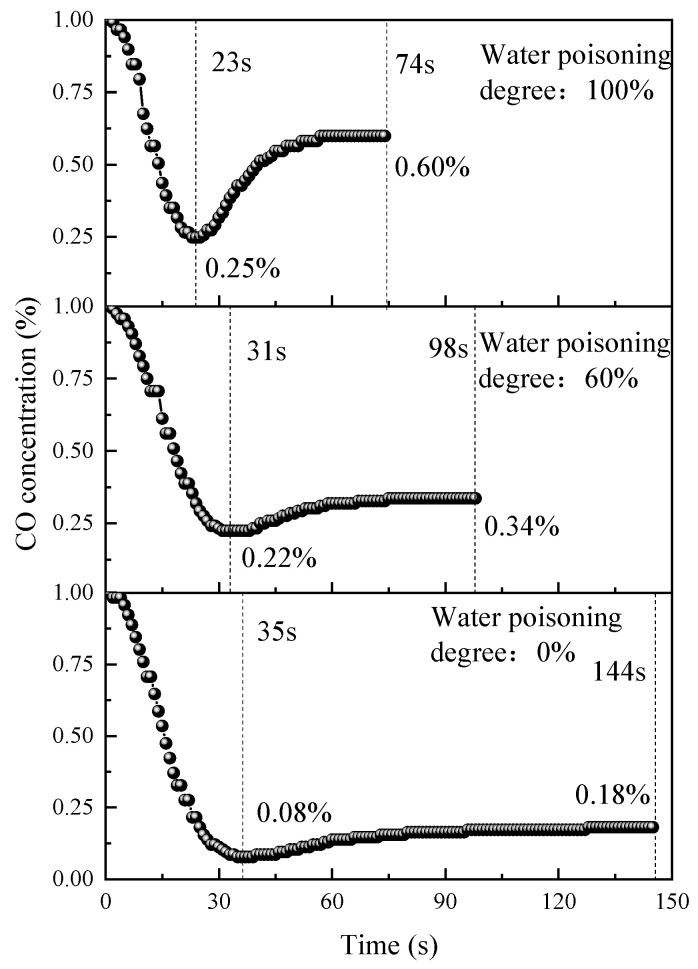
Variation in the CO concentration during the reaction for catalysts with different water poisoning degrees.

To investigate the effects of different water poisoning degrees (0%, 60%, and 100%) on catalyst performance, the CO concentration changes during reactions were monitored, and the results are shown in Figure 14.

As shown in Figure 14, all three catalysts exhibited similar CO concentration variation trends, with the CO concentration rapidly decreasing to minimum points in the initial reaction period. Compared with the unpoisoned catalyst, 60% and 100% water-poisoned catalysts showed elevated minimum CO concentrations and shortened reaction processes. As the poisoning degree increased, the minimum CO concentration further increased, with times to reach minimum points advancing by 4 s and 8 s, respectively. This is mainly because H_2_O molecules occupied active sites on the catalyst surface, reducing CO adsorption sites and thereby decreasing catalytic performance.

Compared with the 0% water poisoning sample, the 60% and 100% water-poisoned samples reached reaction equilibrium in shorter timeframes, with equilibrium times advancing by 46 s and 74 s, respectively. However, the elimination equilibrium concentration increased as the water poisoning degree increased, with the equilibrium concentrations increasing by 0.14% and 0.42%, indicating that water poisoning is unfavorable for CO elimination using the catalyst. This is because after H_2_O molecules occupy catalyst surface active sites during the water poisoning process, the catalyst surface’s ability to adsorb CO and O_2_ weakens. From the overall reaction perspective, a higher water poisoning degree means that there are fewer CO and O_2_ molecules adsorbed on the catalyst surface’s active sites, but the reaction of adsorbed CO and O_2_ proceeds normally. Therefore, a higher water poisoning degree results in shorter catalytic oxidation equilibrium times but elevated equilibrium concentrations, i.e., poorer CO elimination effectiveness.

**Figure 15:**
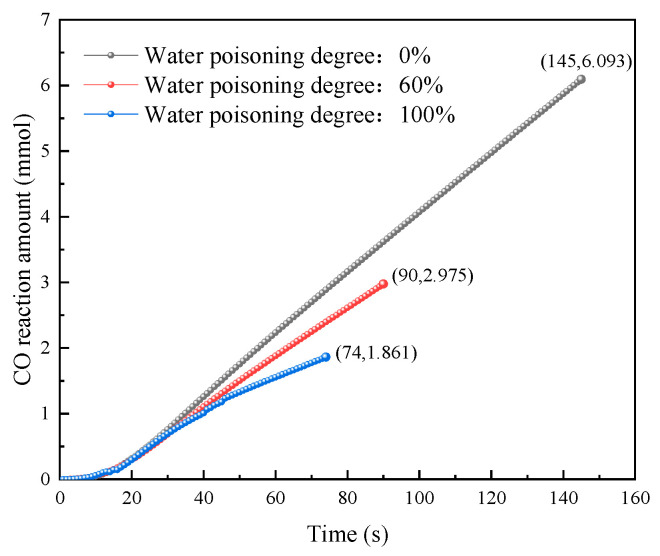
Variation in the CO reaction amount with time at different water poisoning degrees.

**Figure 16:**
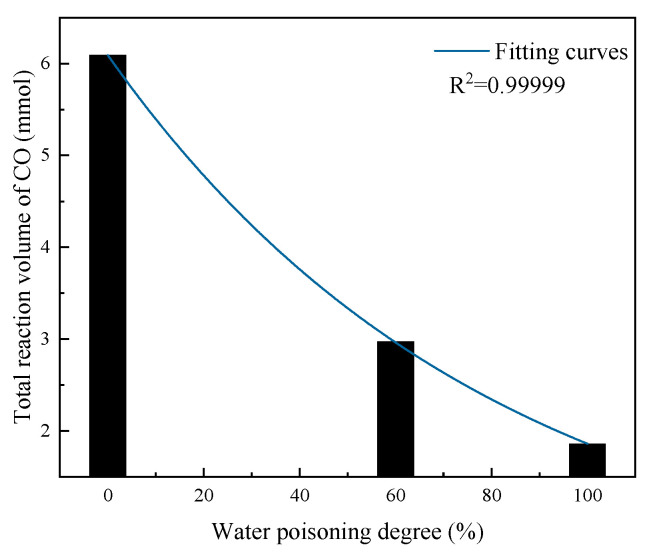
Total elimination reaction amount at different water poisoning degrees.

To further analyze CO reaction amounts during reactions under different water poisoning conditions, the CO reaction amount versus time curves and total CO reaction amount versus water poisoning degree curves were plotted, as shown in Figure 15 and Figure 16.

As shown in Figure 15, at catalyst water poisoning degrees of 0%, 60%, and 100%, the CO reaction amount variation trends with time were generally similar, with slow increases in the early reaction period and essentially linear increases in the later period. In the early reaction period, the CO reaction amounts for 0%, 60%, and 100% water poisoning degrees essentially overlapped. This is because, even though H_2_O molecules occupied some catalyst active sites during the water poisoning process, the active sites remained in an oversaturated state relative to CO molecules in the early reaction period, with little effect on early CO elimination. As reaction time progressed, catalyst surface active sites gradually became occupied. Since a higher catalyst water poisoning degree means fewer clean active sites on the surface, catalyst surface active sites were more easily completely occupied. In the later reaction period, the CO reaction amounts showed obvious differences, with the order of 0% > 60% > 100%.

As shown in Figure 16, the total CO reaction amount exhibited exponential decay with an increasing water poisoning degree. At catalyst water poisoning degrees of 0%, 60%, and 100%, the total CO reaction amounts were 6.09 mmol, 2.97 mmol, and 1.86 mmol, respectively. When the catalyst was completely water-poisoned, the total CO reaction amount decreased by 69.46% compared with the unpoisoned catalyst, indicating that the water poisoning degree has a very significant effect on the catalyst elimination performance. This is mainly because the water poisoning process affects the first step of the catalytic oxidation reaction, hindering the chemisorption of CO and O_2_ molecules on the catalyst surface, thereby hindering the CO catalytic oxidation process and reducing catalyst elimination performance.

#### 2.4.2. Effect of Water Poisoning on Catalyst Activity

**Figure 17:**
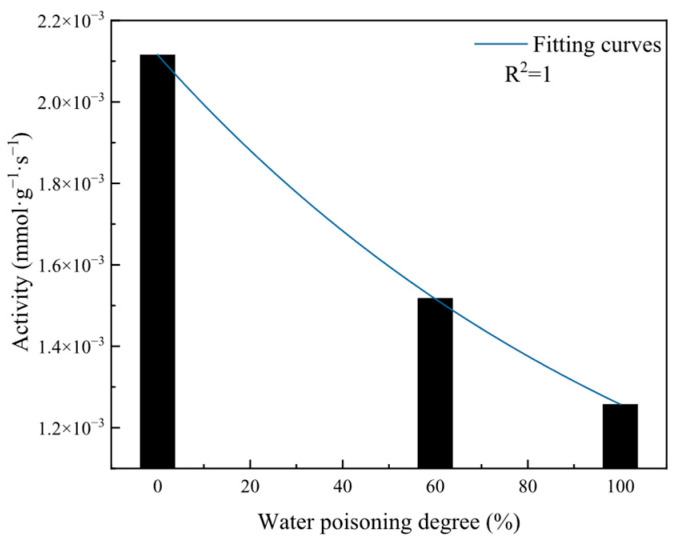
Effect of different water poisoning degrees on catalyst activity during reaction.

**Figure 18:**
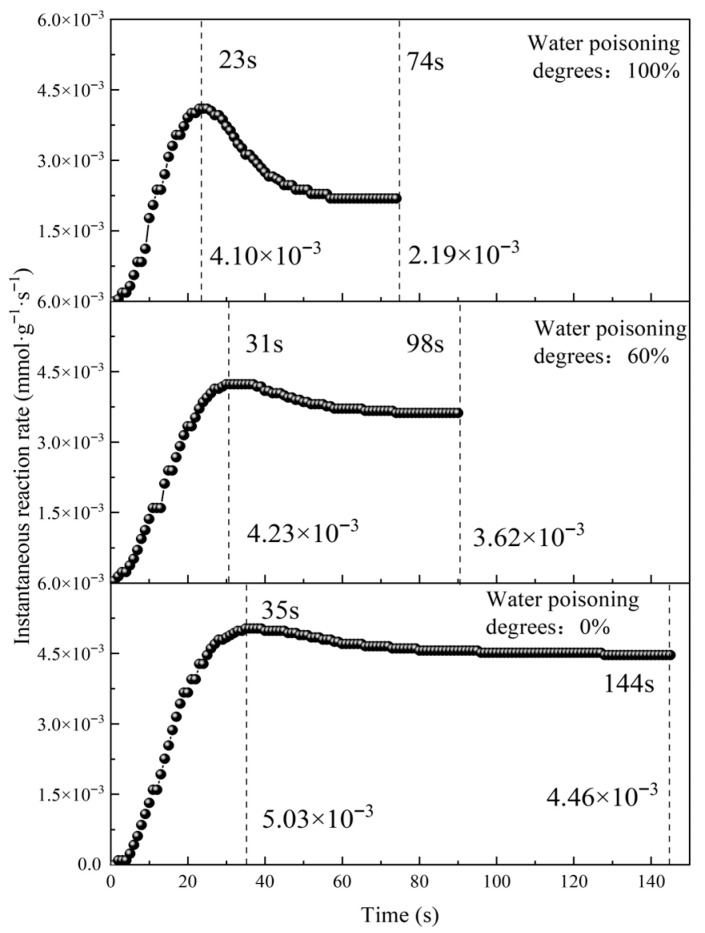
Variation in the instantaneous reaction rate with time at different water poisoning degrees.

To obtain the effect of the water poisoning degree on the catalyst’s CO elimination activity, total catalyst activities and instantaneous reaction rates at water poisoning degrees of 0%, 60%, and 100% were calculated, and the curves are plotted in Figure 17 and Figure 18.

As shown in Figure 17, at water poisoning degrees of 0%, 60%, and 100%, the catalyst activities were 2.12 × 10^−3^ mmol·g^−1^·s^−1^, 1.52 × 10^−3^ mmol·g^−1^·s^−1^, and 1.26 × 10^−3^ mmol·g^−1^·s^−1^, respectively. The catalyst activity exhibited exponential decay with an increasing water poisoning degree. This is because as the water poisoning degree increases, more H_2_O molecules occupy catalyst surface active sites, leaving fewer clean active sites on the catalyst surface. The difficulty of adsorbing CO and O_2_ molecules on catalyst surface active sites increases, the adsorption rate correspondingly decreases, and the total reaction rate also slows down with an increasing water poisoning degree. Therefore, catalysts with higher water poisoning degrees have lower total activity during the reaction process.

As shown in Figure 18, catalysts with different water poisoning degrees showed generally similar instantaneous reaction rate variation trends: they all rapidly increased in the early reaction period, slowly decreased after reaching maximum instantaneous reaction rates, and finally reached catalytic oxidation equilibrium with unchanged instantaneous reaction rates. The water poisoning degree significantly affected the catalyst’s instantaneous reaction rates, especially the maximum reaction rates. Since the effect of the water poisoning degree on catalyst elimination performance primarily affects CO and O_2_ adsorption rates, this indicates that the adsorption process plays an important role in the overall catalytic oxidation process.

At water poisoning degrees of 0%, 60%, and 100%, maximum instantaneous reaction rates were 5.03 × 10^−3^ mmol·g^−1^·s^−1^, 4.23 × 10^−3^ mmol·g^−1^·s^−1^, and 4.10 × 10^−3^ mmol·g^−1^·s^−1^, respectively. The maximum instantaneous reaction rate exhibited exponential decay with an increasing water poisoning degree. This is because a higher water poisoning degree results in more H_2_O occupying catalyst surface active sites, a weakened ability to adsorb CO and O_2_, and a reduced elimination ability, causing the maximum instantaneous reaction rate to gradually decrease with the increase in the water poisoning degree. The 60% water-poisoned catalyst showed a 15.9% decrease in the maximum instantaneous reaction rate compared with the 0% water-poisoned catalyst, while the 100% water-poisoned catalyst showed only a 3.07% decrease compared with the 60% water-poisoned catalyst. This indicates that the water poisoning degree has a more significant effect on catalyst performance in the 0–60% range, representing a critical interval for performance decline. Therefore, water poisoning should be avoided as much as possible during catalyst use.

#### 2.4.3. Effect of Water Poisoning on Catalyst Elimination Efficiency

**Figure 19:**
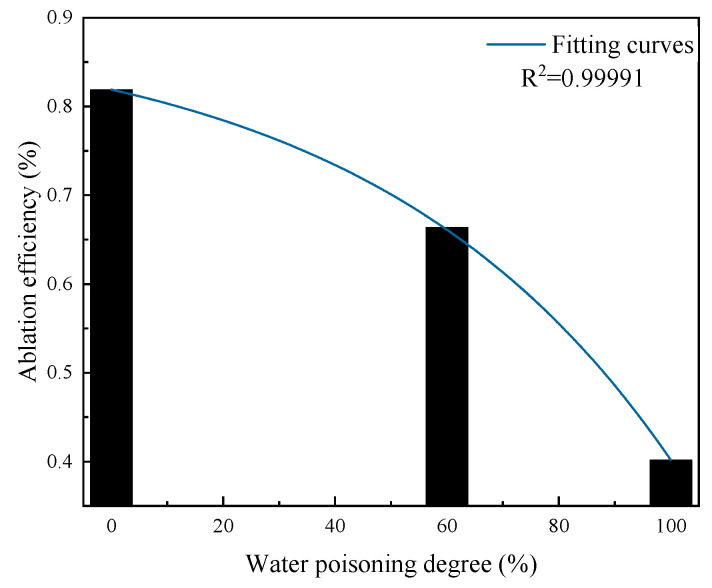
Variation in the elimination efficiency with water poisoning degree.

Figure 19 presents the variation pattern of elimination efficiency with the water poisoning degree. At water poisoning degrees of 0%, 60%, and 100%, the elimination efficiencies were 81.90%, 66.38%, and 40.17%, respectively. As the catalyst water poisoning degree increased, the catalyst elimination efficiency exhibited exponential decline. This is because after catalyst water poisoning, H_2_O molecules occupy catalyst surface active sites, leading to fewer clean active sites on the catalyst surface and weakened adsorption ability for CO and O_2_, thus reducing catalyst elimination efficiency after water poisoning.

#### 2.4.4. Effect of Water Poisoning on Catalyst Stability

**Figure 20:**
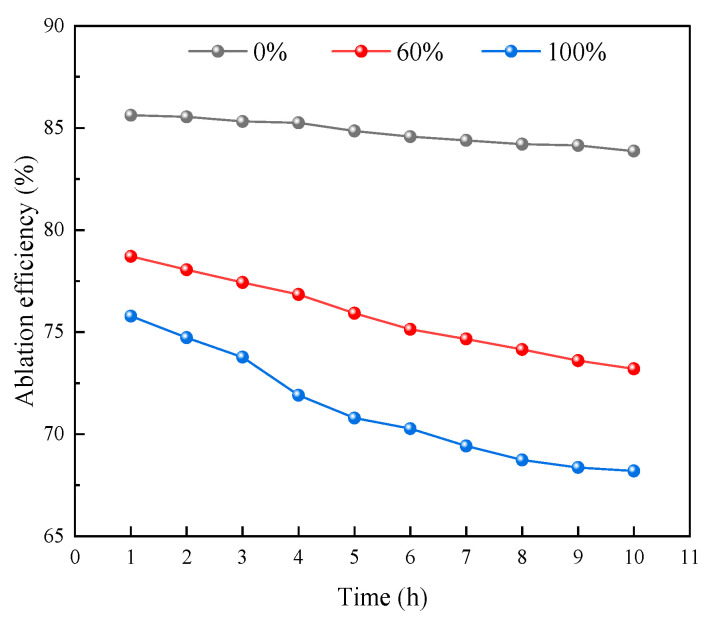
Stability testing for samples with different water poisoning degrees.

**Figure 21:**
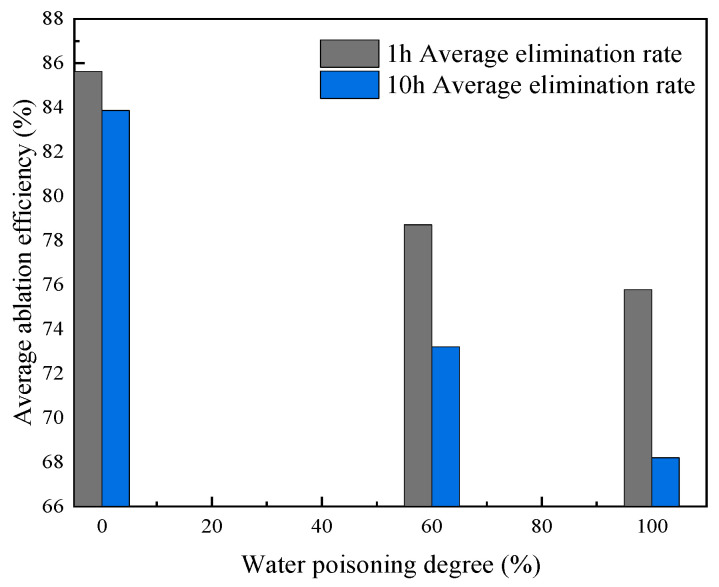
Effect of water poisoning degree on 10 h average elimination efficiency.

In addition to affecting catalyst activity, sample water poisoning also has certain effects on stability. Sample stability at different water poisoning degrees was evaluated in terms of CO elimination efficiency. Figure 20 shows the results of stability analysis obtained via calculating the average CO elimination efficiency every hour for 10 h at a constant temperature of 25 °C and flow rate of 80 mL/min. Figure 21 shows the 10 h average elimination efficiency for samples with different water poisoning degrees.

As shown in Figure 20, in the initial experimental period, all samples maintained relatively high elimination rates. At water poisoning degrees of 0%, 60%, and 100%, the average CO elimination rates after 1 h of elimination were 85.62%, 78.71%, and 75.78%, respectively; these elimination rates decreased sequentially as the water poisoning degree increased, indicating that the water poisoning degree significantly affects the sample elimination rate. As the reaction proceeded, samples with different water poisoning degrees showed varying degrees of decline after 10 h of experimentation.

As shown in Figure 21, at water poisoning degrees of 0%, 60%, and 100%, the average elimination rates after 10 h were 83.87%, 73.2%, and 68.2%, respectively, representing decreases of 1.75%, 5.51%, and 7.58% compared to the 1 h average elimination efficiencies. A higher water poisoning degree resulted in lower 10 h average elimination efficiency, greater decline in elimination efficiency, poorer CO elimination ability, and worse stability. The reason for decreased stability may be that generated CO_2_ combines with water to form carbonate, which covers CO or O_2_ active sites on the catalyst surface, causing CO’s elimination ability to decline.

## 3. Materials and Methods

### 3.1. Catalyst Preparation

During the preparation of the copper–manganese–tin composite oxide catalyst, Sn does not participate in the catalytic oxidation reaction [33]. In addition, the copper–manganese catalyst exhibits optimal ablation performance when the mass ratio of Cu to Mn is 1:2; therefore, the Cu/Mn mass ratio was maintained at 1:2 during the synthesis of the copper–manganese–tin composite oxide catalyst. Furthermore, Sun et al. reported that the copper–manganese–tin composite oxide catalyst achieves maximum CO abatement efficiency when the mass fraction of Sn is 20% [34,35]. Specifically, the Mn-Sn catalyst with 20% Sn content exhibits a catalytic activity 3.23 times higher than that of the Cu-Mn catalyst. This enhancement is attributed to multiple factors. First, the catalyst achieves the highest specific surface area at 20% Sn content. In addition, X-ray diffraction (XRD) and X-ray photoelectron spectroscopy (XPS) reveal the formation of a CuO phase in the Cu-Mn-Sn sample, accompanied by a lower crystallinity of Cu_14_Mn_15_O_4_. Furthermore, Fourier transform infrared (FTIR) spectroscopy indicates that Sn doping suppresses the adsorption of water on the scavenger surface, and the amounts of adsorbed lattice water and coordinated water are minimized at 20% Sn. These multiple factors collectively demonstrate that the incorporation of Sn exerts a positive effect on the catalytic performance of the catalyst. On this basis, the primary components of the catalyst are Cu, Mn, and Sn, with a mass ratio of 27:53:20.

The catalyst was prepared primarily using the co-precipitation method. Aqueous solutions of Cu(NO_3_)_2_·3H_2_O (0.58 mol/L), Mn(NO_3_)_2_ (50 wt.%), and SnCl_4_·5H_2_O (0.16 mol/L) were mixed at a Cu:Mn:Sn molar ratio of approximately 5:12:2 (corresponding to metal mass fractions of approximately 27:53:20), added dropwise to Na_2_CO_3_ aqueous solution maintained at 70 °C water bath temperature, and then stirring occurred continuously. According to the acidity or alkalinity of the mixed solution, dilute nitric acid or Na_2_CO_3_ solution was used to stabilize the pH at approximately 8.3. After the pH meter reading stabilized, the solution was maintained at 70 °C in a constant-temperature water bath with continuous thorough stirring for 4 h. The precipitate was filtered using a circulating water vacuum pump and washed with deionized water until the filtrate’s pH approached neutrality to remove residual Na^+^ and NO_3_^−^ ions. The filtered and washed precipitate was dried in a drying oven at 110 °C for 12 h. The dried sample was placed in a crucible and calcined in a box-type resistance furnace at 400 °C for 4 h. The prepared catalyst was designated a Sn-containing catalyst; the catalyst without Sn in the control group was a Sn-free catalyst.

To prepare the water-poisoned catalysts, the catalyst was first placed in a drying oven and dried at 110 °C until its mass no longer changed to remove excess water, and then the catalysts were stored in centrifuge tubes.

**Figure 22:**
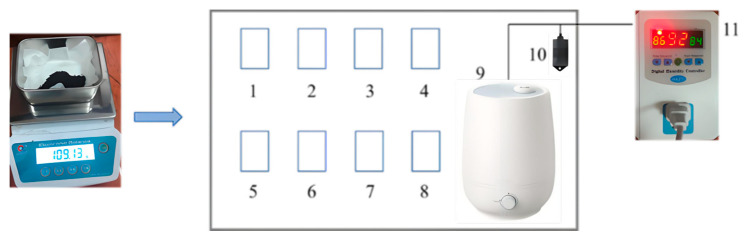
Experimental device.

The experimental setup for the preparation of the sample used in the water resistance test is illustrated in Figure 22. Specifically, 10 g of each sample was accurately weighed using an analytical balance and sequentially placed in stainless steel trays. The trays containing the samples were then put into a sealed chamber equipped with a humidifier, and the humidity inside the chamber was maintained at 95% relative humidity (RH) using a humidity controller (Manufactured by Xiaoxiong Electrical Appliances Co., Ltd., Guangdong, China). The mass of each sample was measured at regular intervals until a constant mass was achieved, after which the samples were taken out and transferred into centrifuge tubes. Among the components, Nos. 1–8 denote the sample-loaded trays, No. 9 represents the humidifier, No. 10 is the humidity sensor, and No. 11 refers to the humidity controller.

The water poisoning degree was calculated according to Equation (1):

(1) $$w=\frac{m_{Water}}{m_{water,sat}}\times 100\%$$

where *w* is the water poisoning degree (%); $m_{Water}$ is the mass of water absorbed by the sample during the water poisoning process (g); and $m_{water,sat}$ is the mass of water absorbed when the sample is completely water-poisoned (g).

### 3.2. Catalyst Activity Testing

**Figure 23:**
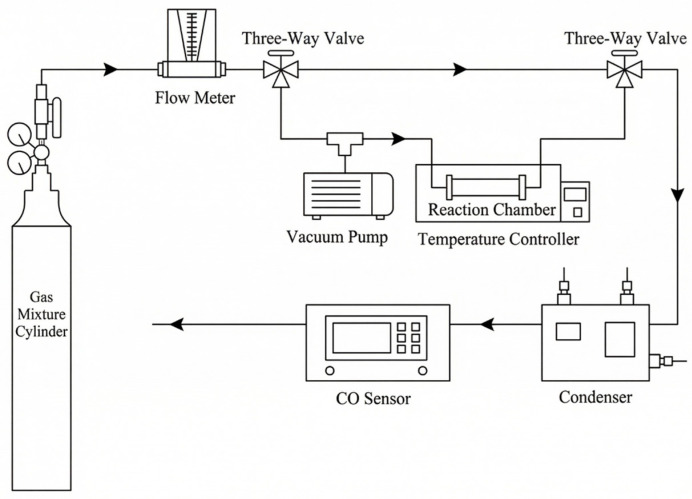
Schematic diagram of the activity evaluation experimental system.

Testing of catalyst activity was conducted using the dynamic method. The activity evaluation experimental system is shown in Figure 23. CO and O_2_ from mixed gas cylinders served as the reaction gases, with N_2_ as the balance gas. The mixed gas flow rate was 80 mL/min (unless otherwise specified, the mixed gas O_2_ concentration was 19.8%, with the CO concentration adjusted within the 1–7% range according to the experimental requirements). The reaction chamber was filled with 10 g of catalyst, and the reaction temperature was controlled by adjusting the constant temperature of the water bath; the temperature was maintained at 25 °C. The condenser cooled and dried the gas to 5 ± 1 °C, and after analysis, the gas was discharged to the atmosphere. All experiments were repeated three times, and the results were averaged. The relative standard deviations were all less than 5%.

For the experimental results, activity, CO reaction amount, instantaneous reaction rate, and elimination efficiency were used to characterize the CO catalytic oxidation performance of the catalyst.

1. Activity

In catalytic reactions, the activity level indicates the strength of the catalyst’s acceleration effect on the reactions, with catalyst activity being the catalytic reaction rate. The activity per unit mass of the catalyst was used as the optimization index. The reaction rate was defined by Equation (2), where $\overset{\cdot }{\xi }$ is the reaction rate, ξ is the reaction extent, and t is time:

(2) $$\overset{\cdot }{\xi }≝\frac{d\xi }{dt}$$

The reaction extent ξ was defined using Equation (3), where $\Delta n_{B_{i}}$ is the change in the amount of component $B_{i}$ (unit: mol), and $v_{i}$ is the stoichiometric coefficient of component $B_{i}$ (positive for products, negative for reactants):

(3) $$d\xi ≝\frac{\Delta n_{B_{i}}}{v_{i}}$$

According to the ideal gas law shown in Equation (4), where p is pressure (unit: Pa), V is gas volume (unit: m^3^), n is the amount of substance (unit: mol), T is temperature (unit: K), and R is the molar gas constant, taken as 8.314472 J·mol^−1^·K^−1^,

(4) pV=nRT

The change in the amount of component $B_{i}$ is shown in Equation (5), where $n_{B_{i}}$ and $n_{B_{i}}^{0}$ are amounts of substance *i* at time *t* and *t* = 0, respectively (unit: mol), and $V_{B_{i}}$ is the instantaneous volume change in CO during the reaction (unit: m^3^):

(5) $$\Delta n_{B_{i}}=n_{B_{i}}-n_{B_{i}}^{0}=\frac{P}{R\cdot T}\cdot \int_{0}^{t}V_{B_{i}}dt$$

The instantaneous volume change is shown in Equation (6), where *L* is the mixed gas flow rate (unit: mL/min), c is the measured instantaneous CO concentration (unit: %), $c_{0}$ is the initial CO concentration in mixed gas (unit: %), and t is time to reach reaction equilibrium (unit: s):

(6) $$V_{B_{i}}=\frac{L}{60}\cdot \left(c-c_{0}\right)\cdot t\cdot 10^{-6}$$

In catalytic reaction systems, if non-catalytic reaction rates are negligible, catalyst activity is calculated as shown in Equation (7), where s is catalyst activity (unit: mol·g^−1^·s^−1^) and m is catalyst mass (unit: g):

(7) $$s=\frac{1}{m}\cdot \frac{d\xi }{dt}$$

2. CO reaction amount

The CO reaction amount is the amount that CO changes by during the reaction process. Based on Equation (3), integrating the amount of CO gas over time provides the CO reaction amount, as shown in Equation (8), where $\Delta n_{CO}\left(t\right)$ is the CO reaction amount (unit: mmol):

(8) $$\Delta n_{CO}\left(t\right)=\frac{P\cdot 10^{-6}}{R\cdot T}\cdot \int_{0}^{t}V_{B_{i}}dt$$

3. Instantaneous Reaction Rate

The instantaneous reaction rate is the amount by which CO changes per unit time per unit mass of catalyst during the reaction process. Differentiating the CO reaction amount with respect to time and dividing by catalyst mass m provides Equation (9), where $v_{CO}\left(t\right)$ is the instantaneous reaction rate (unit: mmol·g^−1^·s^−1^):

(9) $$v_{CO}\left(t\right)=\frac{1}{m}\cdot \frac{d\Delta n_{CO}}{dt}$$

4. Elimination Efficiency

The elimination efficiency is the efficiency of CO elimination during the reaction process, expressed as the ratio of total CO reaction amount to total CO input during the experiment. The calculation method is shown in Equation (10), where η is the elimination efficiency (unit: %), $n_{co,in}$ is the amount of CO introduced at the inlet during the reaction (unit: mmol), and $n_{co,out}$ is the amount of CO at the outlet during the reaction (unit: mmol):

(10) $$\eta =\frac{n_{co,in}-n_{co,out}}{n_{co,in}}\times 100\%$$

## 4. Conclusions

Combining typical underground coal mine environmental factors, this study systematically investigated the effects of the CO concentration, reaction temperature, and water poisoning degree on the CO oxidation performance of a CuMnSn catalyst. The results demonstrated that within the 1–7% concentration range, the catalyst could reduce the CO concentration to below 0.55% within 248 s, exhibiting the capability to induce a rapid emergency response. The catalyst’s activity and maximum instantaneous reaction rate both increased linearly as the CO concentration increased, indicating kinetic advantages in treating high-concentration CO. Elevated temperatures significantly shortened the time it took to reach reaction equilibrium and reduced the equilibrium concentration, with elimination efficiency reaching 99.99% at 300 °C. However, limited by the adsorption step, the catalyst’s activity and maximum instantaneous rate decreased as temperature increased, suggesting the existence of an optimal operating temperature window. Water molecules occupy active sites, causing a comprehensive decline in catalyst activity, elimination efficiency, and long-term stability. The performance decay exhibited an exponential relationship with the water poisoning degree, with the most severe decay in the 0–60% range, where a 60% water poisoning degree can be regarded as the upper limit of the performance-sensitive interval. Therefore, moisture-proof measures or hydrophobic modification of the catalyst should be implemented in practical applications. These findings provide an important basis for evaluating the applicability of CuMnSn catalysts in extreme underground coal mine environments and controlling the adaptability of operating conditions. Additionally, this study preliminarily analyzed the catalytic oxidation process based on dynamic gas concentration changes. Detailed reaction mechanisms await further investigation through in situ spectroscopy and surface characterization techniques.

## Data Availability

The data presented in this study are available on request from the corresponding authors.
